# Utility of ascitic tumor markers and adenosine deaminase for differential diagnosis of tuberculous peritonitis and peritoneal carcinomatosis

**DOI:** 10.1186/s12876-022-02480-x

**Published:** 2022-09-17

**Authors:** Li Du, Xiuqi Wei, Zhuanglong Xiao, Hui Wang, Yuhu Song

**Affiliations:** 1 Division of Gastroenterology, Union Hospital, Tongji Medical College, Huazhong University of Science and Technology, Wuhan, 430022 China; 2 Department of Clinical Laboratory, Union Hospital, Tongji Medical College, Huazhong University of Science and Technology, Wuhan, 430022 China

**Keywords:** Tuberculous peritonitis, Peritoneal carcinomatosis, Ascitic tumor markers, Ascitic adenosine deaminase

## Abstract

**Background:**

Differential diagnosis between tuberculous peritonitis and peritoneal carcinomatosis remains challenging in clinical practice; thus, in-patients diagnosed with tuberculous peritonitis or peritoneal carcinomatosis were retrospectively enrolled, and diagnostic values of ascitic tumor markers and adenosine deaminase were determined.

**Methods:**

Consecutive patients diagnosed with tuberculous peritonitis or peritoneal carcinomatosis were retrospectively enrolled. The pertinent data of 169 patients enrolled were collected.

**Results:**

A panel of ascitic tumor makers (CEA, CA15-3, CA19-9) had high specificity (96.83%) and accuracy (94.67%) in the differentiation of peritoneal carcinomatosis from tuberculous peritonitis; and ascitic ADA was a good discriminator between these patients, with an accuracy of 91.72%. Combined use of ascitic tumor makers and ADA (ascitic ADA < 22.5 IU/L or ascitic CEA > 3.65 ng/mL or CA15-3 > 42.70 U/mL or CA19-9 > 25.10 U/mL) performed high sensitivity (99.06%) and accuracy (94.08%) for the diagnosis of peritoneal carcinomatosis. In addition, combined ascitic ADA and tumor marker (positive ascitic tumor makers and ADA < 22.50 IU/L) had 100% of the specificity in diagnosing peritoneal carcinomatosis.

**Conclusions:**

Combined use of ascitic tumor markers and adenosine deaminase showed excellent efficiency in the differential diagnosis between tuberculous peritonitis and peritoneal carcinomatosis, thus these two simple and cost‐effective parameters should be determined when tuberculous peritonitis or peritoneal carcinomatosis was suspected in clinic practice.

**Supplementary Information:**

The online version contains supplementary material available at 10.1186/s12876-022-02480-x.

## Background

Tuberculous peritonitis (TBP) and peritoneal carcinomatosis (PC) are two of the most common causes of non-portal hypertensive ascites in developing countries [1, 2], and both diseases require accurate recognition for the appropriate management [3, 4]. Our previous studies have illustrated ascitic cholesterol and total protein were excellent measures in distinguishing non‐portal hypertensive ascites from portal hypertension (PH) [2, 5, 6]. However, tuberculous ascites and malignant ascites have similar clinical profiles, thus the differential diagnosis remains challenging [7–9].

Laparoscopic peritoneal biopsy is considered to be an excellent choice in distinguishing TBP from PC [10], but its clinical application is limited because this procedure is invasive for the patients. Previous studies have illustrated ascitic ADA provided an assistance in differential diagnosis of TBP or non-TBP related ascites [11–13]. However, besides TBP patients, some of patients with secondary bacterial peritonitis also have high ascitic ADA level [14], which diminished the accuracy of ascitic ADA in diagnosing TBP. In addition, the relatively small sample size was another factor that confined the clinical application of ascitic ADA [11–13]. Seung et al. has used a small sample (27 cases of TBP and 25 cases of PC) to indicate that, ascitic fluid ADA measurement showed excellent differential value between TBP and PC [12]. In addition, we previously demonstrated that a panel of ascitic tumor markers yielded high accuracy in the differentiation of malignant ascites from benign ascites [15]. In this study, a larger number of the patients with TBP or PC were retrospectively enrolled, and then differentiating value of combined ascitic tumor markers and ADA were determined.

## Methods

### Patient selection and diagnosis criteria

In this retrospective cohort study, patients over 18 years old with new‐onset ascites who were admitted to Union Hospital of Huazhong University of Science and Technology (Wuhan, China) were assessed for eligibility from May 2015 to January 2022. All patients had laboratory tests such as peripheral blood count, serum biochemical tests, ascitic cell count and biochemical tests, and ascitic tumor marker assay. The diagnosis criteria of TBP was based on the biopsy of the peritoneal nodules, or complete clinical and laboratory response after anti-tuberculous therapy when other causes of ascites were excluded [5]. Peritoneal carcinomatosis was referred to positive cytology in peritoneal fluid, or positive peritoneal biopsy, or primary malignancy after ruling out benign etiologies of the ascites [5]. For cytologically positive malignant ascites, malignant ascites with unknown primary carcinoma referred to the patients with unspecified origin [15]. For malignant ascites with negative cytology or without cytological examination, a diagnosis of unspecified primary was made in the patients with a known malignancy which involved two or more organs after ruling out other causes [15]. It was confirmed by radiological finding and/or histological examination. Patients were excluded if they received antituberculosis therapy before the detection of tuberculous peritonitis. The study was conducted according to the principles of the Declaration of Helsinki, and the protocol was approved by Ethics Committee of Tongji Medical School, Huazhong University of Science and Technology.

### Ascitic tumor marker assay

Ascites samples obtained by paracentesis were collected in tubes, and then sent for tumor marker assay. Carcinoembryonic antigen (CEA), cancer antigen CA15-3 and CA19-9 were tested on Abbott i2000 by the chemiluminescence method using manufacture’s chemiluminescent immunoassay reagents (Abbott, Chicago, IL).

### Ascitic adenosine deaminase assay

Ascites samples obtained by paracentesis were collected in tubes, and then sent for biochemical assay. The ascitic adenosine deaminase was determined by the peroxidase techniques using adenosine deaminase determination assay kit (Beijing Leadman Biochemistry Co., Ltd, Beijing, China).

### Statistical Analysis

All statistical analyses were conducted using the SPSS statistical software (version 23.0; IBM, Armonk, NY), with *p* < 0.05 considered statistically significant. Independent-samples non-parametric test was used for the analysis of differences between the two groups. The cut-off values of continuous variables for differentiation between the groups were determined based on receiver operating characteristic (ROC) analysis. Sensitivity, specificity, diagnostic accuracy, positive predictive values (PPVs), and negative predictive values (NPVs) were calculated with cut-offs defined by choosing the largest Youden index.

## Results

### Clinical characteristics of patients

A total of 169 patients were enrolled for this study, 63 patients were diagnosed with tuberculous peritonitis and 106 patients with peritoneal carcinomatosis. In patients with peritoneal carcinomatosis, the etiological distribution was presented in Additional file 1: Table S1. The clinical characteristics of the enrolled patients were shown in Table 1.Firstly, the data of demographic parameter showed the onset age of TBP patients was significantly earlier than that of PC patients [44.00 *vs* 60.00, *p* < 0.0001], which was consistent with previous study [16]. And the mean arterial pressure was lower in TBP patients [94.75 *vs* 98.50 mmHg, *p* < 0.01]. Secondly, we collected the data on the biomarkers of liver or kidney function, such as alanine aminotransferase, aspartate aminotransferase, alkaline phosphatase, γ-glutamyl transpeptidase, creatinine and uric acid, and there was no significant difference between the two groups.

**Table 1:** Demographic characteristics and the results of clinical biochemistry in TBP and PC

Parameter	Tuberculous peritonitis	Peritoneal carcinomatosis	*p* value	Total Cohort
Age, years	44.00 (28.00–53.00)	60.00 (48.00–69.00)	< 0.0001	53.00 (40.00–67.00)
Gender, *n* (M/F)	28/35	45/61	0.8728	73/96
Heart rate, bpm	78.00 (76.00–82.50)	78.00 (78.00–88.00)	0.3041	78.00 (78.00–84.00)
Mean arterial pressure, mmHg	94.75 (84.50–100.00)	98.50 (93.00–107.30)	0.0060	97.50 (89.38–104.00)
**Clinical biochemistry**				
Total bilirubin, μmol/L	8.70 (6.25–12.50)	10.40 (7.63–13.63)	0.1911	10.00 (7.03–12.85)
Conjugated bilirubin, μmol/L	3.20 (2.43–5.70)	3.45 (2.03–5.18)	0.9454	3.40 (2.30–5.45)
Alanine aminotransferase, U/L	14.00 (10.00–21.50)	13.00 (10.00–19.50)	0.3852	14.00 (10.00–20.00)
Aspartate aminotransferase, U/L	19.00 (16.25–25.75)	21.50 (17.00–28.75)	0.4734	21.00 (17.00–26.25)
Alkaline phosphatase, U/L	69.00 (53.00–80.00)	71.00 (57.00–88.00)	0.2227	69.50 (56.25–85.75)
γ-glutamyl transpeptidase, U/L	21.00 (14.00–29.00)	22.00(14.00–37.00)	0.5625	21.50 (14.25–32.00)
Serum total protein, g/L	63.60 (59.40–66.60)	61.05(56.43–66.25)	0.1595	62.50 (57.20–66.40)
Serum albumin, g/L	34.25 (31.20–36.93)	34.60 (31.03–37.98)	0.6136	34.30 (31.08–37.73)
Urea, mmol/L	4.26 (3.15–5.08)	5.43 (4.00–7.29)	0.0029	4.88 (3.47–6.24)
Creatinine, µmol/L	61.30 (54.78–71.58)	65.60 (55.70–77.10)	0.1468	63.90 (55.40–75.45)
Uric Acid, µmol/L	279.60 (238.00–341.20)	327.90(244.60–401.30)	0.1547	298.90 (243.25–384.90)
**Ascitic fluid analysis**				
Ascitic total protein, g/L	50.30 (43.15–56.08)	45.30 (40.70–50.85)	0.0087	48.40(42.10–53.75)
Ascitic albumin, g/L	29.70 (26.63–31.45)	29.30 (26.35–32.65)	0.9817	29.40 (26.80–32.15)
SAAG, g/L	4.00 (2.70–7.55)	5.75 (1.95–9.80)	0.4625	4.55 (2.48–8.13)
Ascitic cholesterol, mmol/L	2.31 (1.97–2.61)	2.50 (2.10–3.12)	0.0420	2.44 (2.08–2.86)
Ascitic ADA, IU/L	35.00 (28.00–48.00)	8.25 (6.00–11.40)	< 0.0001	11.20 (7.00–32.00)
Ascitic CEA, ng/mL	0.90 (0.60–1.10)	179.90 (1.90–143)	< 0.0001	3.20 (0.80–661.53)
Ascitic CA15-3, U/mL	19.50 (16.05–30.30)	17.35 (5.98–166.00)	0.7809	19.40 (7.85–106.10)
Ascitic CA19-9, U/mL	3.60 (2.00–8.55)	150.10 (10.18–1200.00)	< 0.0001	23.70 (3.00–764.10)
Ascitic, AFP ug/L	1.45 (1.00–1.83)	1.50 (1.00–2.08)	0.5476	1.50 (1.00–2.00)

M/F, male/female. Continuous variables are expressed as median and interquartile range

**Fig. 1:**
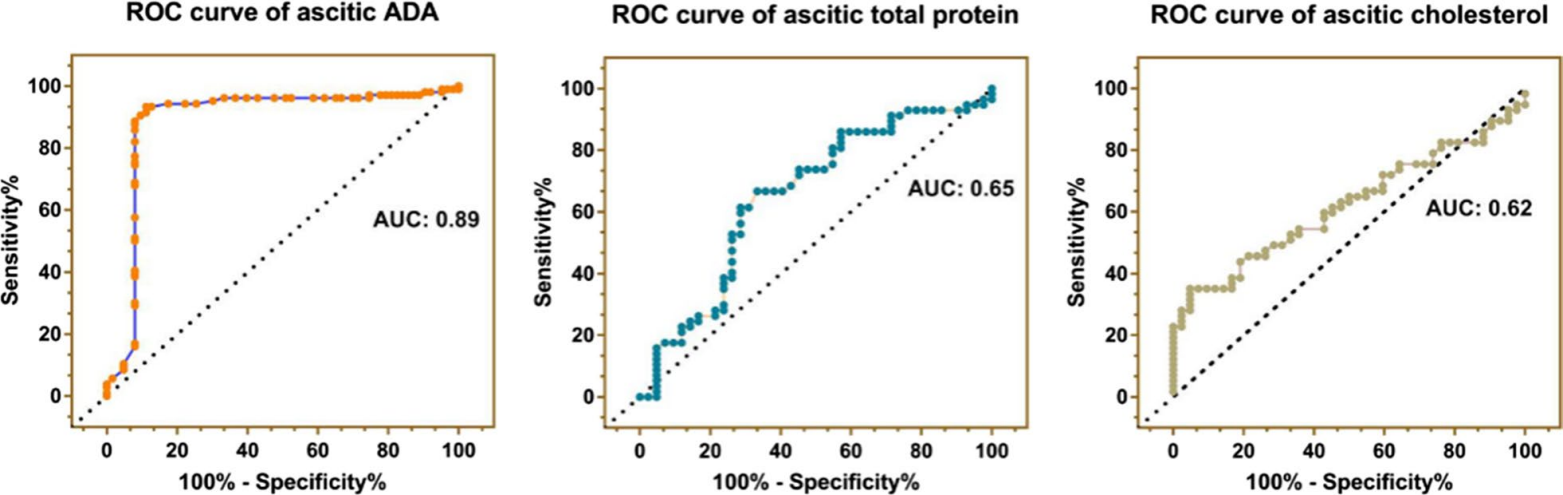
ROC curves of ascitic ADA, ascitic total protein and ascitic cholesterol in differentiating PC from TBP

As for ascitic parameters, the concentration of ascitic total protein [50.30 (43.15, 56.08) g/L vs 45.30 (40.70, 50.85) g/L, *p* < 0.01] and ascitic ADA [35.00 (28.00, 48.00) IU/L vs 8.25 (6.00, 11.40) IU/L, *p* < 0.0001] in TBP were significantly higher than those in PC, with 0.65 and 0.89 of the AUC, respectively (Fig. 1). While ascitic cholesterol in TBP was significantly lower than that in PC [2.31 (1.97, 2.61) mmol/L vs 2.50 (2.10, 3.12) mmol/L, *p* < 0.05], with 0.62 of the AUC (Fig. 1). The above data indicated that the ascitic ADA was a good discriminator for TBP and PC. Finally, the concentration of ascitic CEA and CA19-9 in TBP was significantly lower than those in PC. As for ascitic CA15-3, there was no significant difference in CA15-3 level between TBP and PC, but 42.00% of the PC patients had CA15-3 > 42.70 U/mL while only 4.76% of the TBP patients did (Additional file 1: Fig. S1), suggesting that ascitic CA15-3 was still a specific marker for the differential diagnosis of TBP and PC.

### Ascitic tumor markers had high specificity and accuracy for PC

Tumor markers are produced directly by the tumor or by non-tumor cells as a response to the presence of a tumor, which offers a putative clinical use in the screening, diagnosis and treatment of various cancers [15, 17].Our previous study has demonstrated that, a panel of three different ascitic tumor markers (ascitic CEA > 50.00 ng/mL or CA15-3 > 75.00 U/mL or CA19-9 > 200.00 U/mL) yielded a sensitivity of 85.45% and a specificity of 97.32% in the diagnosis of malignant or benign ascites [15]. In this current study we defined 3.65 ng/mL of the ascitic CEA, 42.70 U/mL of the ascitic CA15-3 and 25.10 U/mL of the ascitic CA19-9 as the cut-off values in the differential diagnosis of TBP and PC, based on the largest Youden index. As a result (shown in Table 2), the combination of three tumor markers yielded a specificity of 96.83%, sensitivity of 93.40%, and accuracy of 94.67% in the diagnosis of PC. Furthermore, positive ascitic CEA alone, CA15-3 alone or CA19-9 alone had specificity of 100.00%, 95.24% and 97.78%, respectively, and sensitivity of 72.82%, 42.00%, 71.74%, respectively. In conclusion, the combined ascitic tumor makers performed high specificity and accuracy for the differential diagnosis of PC and TBP.

**Table 2:** Diagnostic performance of ascitic tumor markers in peritoneal carcinomatosis

Variables	Sensitivity (%)	Specificity (%)	PPV (%)	NPV (%)	Accuracy (%)
Ascitic CEA	72.82	100.00	100.00	63.64	81.58
Ascitic CA15-3	42.00	95.24	95.45	40.82	57.75
Ascitic CA19-9	71.74	97.78	98.51	62.86	81.02
Ascitic CEA + CA15-3 + CA19-9 ( CEA or CA15-3 or CA19-9)	93.40	96.83	98.02	89.71	94.67

Cut-off points for ascitic CEA, CA15-3 and CA19-9 were 3.65 ng/mL, 42.70 U/mL and 25.10 U/mL, respectively. Ascitic CEA + CA15-3 + CA19-9 meant ascitic CEA > 3.65 ng/mL, or CA15-3 > 42.70 U/mL, or CA19-9 > 25.10 U/mL

### Ascitic ADA was a good discriminator in TBP and PC

**Fig. 2:**
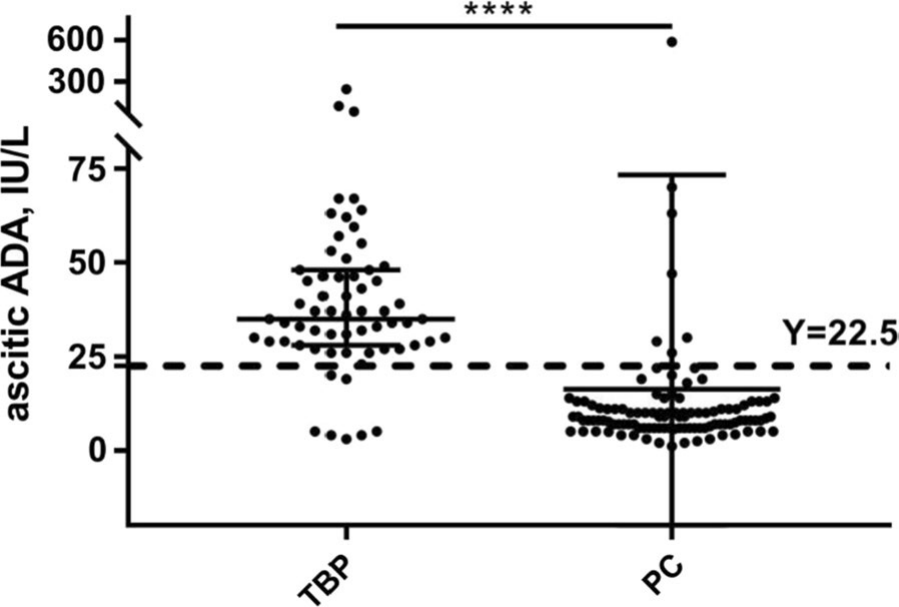
Scatter dot plot showing the distribution of ascitic ADA in the patients enrolled. Median with interquartile range is included, horizontal lines at 22.5 IU/L for ascitic ADA. (****, *p* < 0.0001)

Above data demonstrated the concentration of ascitic ADA in patients with TBP was significantly higher than those with PC (Table 1), then we investigated its differential value between the two groups. The cut‐off value of 22.50 IU/L was chosen in our study based on its largest Youden index. As shown in Fig. 2, high levels of ascitic ADA (≥ 22.50 IU/L) were observed in most TBP patients (88.89%), while most (93.40%) of the PC patients had lower concentrations of ascitic ADA (< 22.50 IU/L). As a result, the ascitic ADA had an accuracy of 91.72% in the total cohort (Table 3). The data above demonstrated that the ascitic fluid ADA was a good discriminator in TBP and PC.

**Table 3:** Diagnostic performance of combined ascitic ADA and tumor markers

Variables	Sensitivity(%)	Specificity (%)	PPV (%)	NPV (%)	Accuracy (%)
ADA (< 22.5 IU/L)	93.40	88.89	93.40	88.89	91.72
ADA + Tumor marker (ascitic ADA < 22.5 IU/L or positive tumor marker)	99.06	85.71	92.11	98.18	94.08
Combining ADA and tumor marker (ascitic ADA < 22.5 IU/L and positive tumor marker)	87.74	100.00	100.00	82.89	92.31

Tumor marker positive meant ascitic CEA > 3.65 ng/mL, or CA15-3 > 42.70 U/mL, or CA19-9 > 25.10 U/mL

### Combined ascitic tumor makers and ADA showed high sensitivity and accuracy in differentiating PC from TBP

Since ascitic tumor makers had high specificity and accuracy in diagnosing PC, and ascitic fluid ADA performed differentiating value in TBP and PC. Next, we determined the diagnostic performance of combined ascitic tumor makers and ADA. As shown in Table 3, the combined ascitic tumor makers and ADA (positive ascitic tumor makers or ADA < 22.50 IU/L) had a sensitivity of 99.06%, specificity of 85.71%, diagnostic accuracy of 94.08%, NPV of 98.18%, and PPV of 92.11% in the diagnosis of PC; while ascitic ADA alone had a sensitivity of 93.40%, specificity of 88.89%, diagnostic accuracy of 91.72%, NPV of 88.89%, and PPV of 93.40%.We concluded that the combined ascitic tumor makers and ADA (positive ascitic tumor makers or ADA < 22.50 IU/L) showed higher sensitivity and accuracy than ADA alone. On the other hand, although the combined ascitic tumor makers and ADA (positive ascitic tumor makers or ADA < 22.50 IU/L) had similar diagnostic accuracy with tumor markers, however, the combination of ascitic tumor makers and ADA (positive ascitic tumor makers or ADA < 22.50 IU/L) showed higher sensitivity for the detection of PC, while the positive tumor markers showed higher specificity. In addition, the combined ascitic ADA and tumor marker (positive ascitic tumor makers and ADA < 22.50 IU/L) had 100% of the specificity in diagnosing PC, which could excellently rule out benign ascites in clinical practice. In conclusion, the combination of ascitic tumor makers and ADA were superior to individual index in clinic practice.

## Discussion

Abdominal paracentesis is likely the most rapid and cost‐effective method of diagnosing the cause of ascites. In this current study we illustrated that ascitic tumor makers had high specificity and accuracy in differential diagnosis of PC from TBP; and ascitic ADA was also a good discriminator in these patients. Combined ascitic tumor makers and ADA were superior to individual index in clinic practice.

Ascitic tumor markers were widely used to differentiate malignant ascites from benign ascites [17–19]. Our previous study found that ascitic CEA, CA15-3 and CA19-9 were valuable in distinguishing benign ascites from malignant ascites [15]. In this current study, we demonstrated that ascitic CEA, CA15-3 and CA19-9 differentiated peritoneal carcinomatosis with tuberculous peritonitis efficiently. However, both the peritoneal carcinomatosis and tuberculous peritonitis were characterized with high level of serum and ascitic CA12-5 [8, 15, 20, 21], thus this parameter was not helpful in the differentiation of peritoneal carcinomatosis from tuberculous peritonitis. Other researchers found ascitic CA72-4 was also valuable in the differential diagnosis of peritoneal carcinomatosis and tuberculous peritonitis [22]. In addition, peritoneal carcinomatosis arising from different organs were characterized with the elevation of specific tumor markers. For example, colorectal cancer was characterized with high level of ascitic CA19-9, CEA; gynecological cancer mainly had high ascitic CA15-3 [15]. Therefore, the combination of biomarkers possesses better performance than single in this study, which was consistent with previous studies [15, 19].

Our previous study has used cut-off values of 50 ng/mL for ascitic CEA, 75 U/mL for ascitic CA15-3 and 200U/mL for ascitic CA19-9 in diagnosing malignant or benign ascites [15]. In this current study, the cut-off values for ascitic CEA, CA15-3 and CA19-9 were 3.65 ng/mL, 42.70 U/mL and 25.10U/mL respectively, much lower than those of our previous study [15]. The difference was attributed to the following factors. Firstly, the cut-off values in this study were determined by choosing the largest Youden index, which was a reliable method in defining the cut-off value. Secondly, our previous study focused on the differentiation of malignant ascites from benign ascites, a higher cut-off values was set to achieve high specificity in the diagnosis of malignant ascites [15].

In decades, ascites ADA has been proposed as a useful diagnostic test in discriminating TBP from non-TB ascites, but there were also some debates about its clinical application in defining the etiologies of ascites. Donald et al. has indicated that ascitic fluid ADA was insensitive in detecting TBP in the United States [14]. 59% (10/17) of their TBP patients enrolled had liver cirrhosis, which resulted in the bias of the conclusion. Consequently, TBP patients with liver cirrhosis had similar low ascitic ADA concentration with that of portal hypertensive ascites, which decreased diagnostic performance of ascitic ADA. Still in their research, 50% (5/10) of patients with secondary bacterial peritonitis had high level of ascitic ADA, which diminished its specificity in diagnosing TBP. In clinic, the secondary bacterial peritonitis was relatively easily diagnosed by the presence of ascitic fluid neutrophil count of greater than 250/mm^3^, or extravasation of contrast material or peritoneal free air on radiography or computerized tomography, and/or perforation of the intestinal wall demonstrated at surgery [5]. Thus, our current study focused on the differential diagnosis between TBP and PC, which remained a challenge in clinical practice. Finally, Seung et al. defined 21 IU/L of ascitic ADA as the cut-off value in differentiating tuberculous peritonitis (n = 27) from malignant ascites [12]. Ayako et al. set 40 IU/L as the cut-off value in differentiating tuberculous peritonitis (n = 15) from malignant ascites, liver cirrhosis and others [13]. Sample size probably resulted in the different cut-off values; importantly, enrolled patients had different underlying causes, which contributed to the difference in cut-off value.

Interestingly, our study found a relatively large number of patients had ADA < 39 IU/L in the TBP group (Additional file 1: Table S2), then the sensitivity was lower than usually reported [13, 23]. Several factors contributed to the difference. Firstly, Ayako et al. selected the highest ADA value when multiple measurements of ascitic ADA level were performed in a single patient [13]. Secondly, patients with a history of ascites may have received diuretic at home, which might increase the concentration of ascitic ADA. However, in our study, we collected the first value of ascitic ADA for patients with new‐onset ascites.

In addition, racial difference might also lead to different concentration of ascitic ADA.

This study had potential limitations. Firstly, this was a single center and retrospective study, and multicenter prospective study with a larger population should be performed to confirm the conclusion. Secondly, we did not explore the mechanism underlying increased concentration of ascitic ADA in TBP.

In summary, combined use of ascitic tumor makers and ADA showed excellent differential performance between TBP and PC patients. Thus, these two simple and cost‐effective parameters should be determined when TBP or PC was suspected in clinic.

## Supplementary Information

**Additional file 1. Table S1**. The etiology of the patients enrolled in our study.** Figure S1**. The distribution of ascitic CA15-3 in the patients.** Table S2**. Diagnostic performance of combined ascitic ADA (<39 IU/L) and tumor markers.

## Data Availability

The datasets used and/or analyzed during the current study are not publicly available because of the strict management by our department. Data are however available from the corresponding author on reasonable request.

## Abbreviations

TBP: Tuberculous peritonitis
PC: Peritoneal carcinomatosis
ADA: Adenosine deaminase
CEA: Carcinoembryonic antigen
CA15-3: Cancer antigen15-3
CA19-9: Cancer antigen 19-9
ROC: Receiver operating characteristic
PPV: Positive predictive value
NPV: Negative predictive value
AUC: Area under curve
