# Supplementary material for: Utility of ascitic tumor markers and adenosine deaminase for differential diagnosis of tuberculous peritonitis and peritoneal carcinomatosis
Source: BMC Gastroenterol. 2022 Sep 17;22:423. doi: 10.1186/s12876-022-02480-x (PMC9482723; doi:10.1186/s12876-022-02480-x)
Supplement: Supplementary file 1 — Additional file 1. Table S1. The etiology of the patients enrolled in our study. Figure S1. The distribution of ascitic CA15-3 in the patients. Table S2. Diagnostic performance of combined ascitic ADA (<39 IU/L) and tumor markers. [file 12876_2022_2480_MOESM1_ESM.docx]

**The etiology distribution of patients and diagnostic**

**value of ascitic CA15-3**

Li Du ^1^*, Xiuqi Wei^2^*, Zhuanglong Xiao^1^, Hui Wang ^2╇^, Yuhu Song ^1╇^

^1^ Division of Gastroenterology, Union Hospital, Tongji Medical College, Huazhong

University of Science and Technology, Wuhan, China

^2^Department of Clinical Laboratory, Union Hospital, Tongji Medical College, Huazhong University of Science and Technology, Wuhan, China

**Table S1** The etiology of the patients enrolled in our study

| **Etiology** | **Total cases**  **(169)** |
| --- | --- |
| **Tuberculous peritonitis** | **63 (37.28%)** |
| **Peritoneal carcinomatosis** | **106 (62.72%)** |
| Gastric cancer | 22 (13.02%) |
| Colorectal cancer | 15 (8.88%) |
| Pancreatic cancer | 8 (4.73%) |
| Gynecological cancer | 28 (16.57%) |
| Primary liver cancer | 7 (4.14%) |
| Gallbladder cancer | 3 (1.78%) |
| Bladder cancer | 1 (0.59%) |
| Lung cancer | 1 (0.59%) |
| Fibroblastoma | 1 (0.59%) |
| Cancer of unknown primary site | 20 (11.83) |

**Figure S1** The distribution of ascitic CA15-3 in the patients


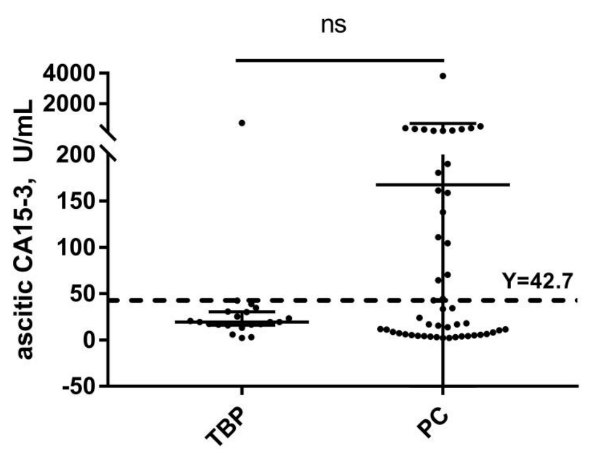


Figure S1: Scatter dot plot showing the distribution of ascitic CA15-3 in the patients enrolled. Median with interquartile range is included, horizontal lines at 42.7 U/mL for ascitic CA15-3.

**Table S2:** **Diagnostic performance of combined ascitic ADA (<39 IU/L) and tumor markers.**

| **Variables** | **Sensitivity (%)** | **Specificity**  **(%)** | **PPV**  **(%)** | **NPV**  **(%)** | | **Accuracy (%)** |
| --- | --- | --- | --- | --- | --- | --- |
| ADA (<39 IU/L) | 96.23 | 41.27 | 73.38 | | 86.67 | 75.74 |
| ADA + Tumor marker (Ascitic ADA< 39 IU/L or positive tumor marker) | 99.06 | 41.27 | 73.94 | | 96.30 | 77.51 |
| Combining ADA and tumor marker (ascitic ADA< 39 IU/L and positive tumor marker) | 90.57 | 96.83 | 97.96 | | 85.92 | 92.90 |

Note: Diagnostic performance of a low ADA (<39 IU/L) in peritoneal carcinomatosis was shown. Tumor marker positive meant ascitic CEA >3.65 ng/mL, or CA15-3 >42.70 U/mL, or CA19-9 >25.10 U/mL
